# Construction Notes

**Published:** 1893-05-13

**Authors:** 


### CONSTRUCTION NOTES.

New Hospital for Falmouth.—Mr. T. Passmore-Edwards laid the foundation-stone of a new hospital for Falmouth, the entire cost of which, estimated at £1.500, he has generously defrayed. Mr. Passmore-Edwards has further stated that if the endowment of the establishment, which is at present £700 or £800, is made up to £1,000, then he will also defray the total cost of the furnishing.

Cardiff Infirmary.—Not only has it been found necessary to extend the accommodation for the ordinary medical and surgical cases at this hospital, but the satisfactory growth of the aural and ophthalmic departments, under the care of Dr. Tatham Thompson, has compelled the committee to make provision for the reception of these cases. The new block in question will extend from the centre of the building southwards, for an extension of the present corridor, and it will be two storeys high, for the reception of male and female patients respectively. In connection with this there is considerable extension of the accommodation for nurses. This accommodation will be obtained on the first floor of the new north and south wings, which will be built out from the present administrative block. The chief portion of the south wing on the ground floor will be occupied by the extension of the out-patients' department, which has also greatly increased in numbers. The ground floor of the north wing will be occupied by a new board-room, with a library attached, a small museum, a dining-room for the staff, and the requisite conveniences in connection therewith. The committee has decided that the removal of the board-room for its present position will be an advantage. Messrs. Seward and Thomas are the architects to whom the work has been entrusted.

New Hospital for Ilkeston.—The foundation-stone was recently laid of this new hospital, which is intended to take the place of the present Cottage Hospital. The site was given by Mr. Mundy; the architect being Mr. C. W. Hunt, of Ilkeston; and the contractor, Mr. W. E. Shaw. The estimated cost is £2,600, of which about £1,700 has been given and promised. The building, as planned, will comprise an extensive, complete, and comprehensive block of buildings, adequate and suitable for the purpose of a well-conducted hospital. The present portion now in course of erection consists of the medical and administrative department, which is two storeys high, the ground floor being planned for the matron's and nurses' bedrooms, children's ward for eight beds, bathroom, &c. Covered corridors are connected to the administrative department with the several wards, the women's ward for six beds being constructed so as to correspond with the laundry wing of the administrative department. The men's ward for twelve beds is constructed in a parallel line with the administrative department, and has a nurses' room thereto. An operating room is provided at the end of the covered corridor. The wards are provided with all necessary bathrooms, &c. A mortuary and other outbuildings are also provided. The whole of the respective buildings will be of Ilkeston red-faced bricks. The roofs will be covered with Broseley tiles. Proper and efficient means of ventilation are provided to each separate ward and bathroom, &c.

